# Supplementary material for: Cell motility influences microfluidics capturing in scRNA-seq
Source: Open Res Eur. 2026 Apr 8;5:154. Originally published 2025 Jun 6. [Version 2] doi: 10.12688/openreseurope.19781.2 (PMC13069381; doi:10.12688/openreseurope.19781.2)
Supplement: Peer review history

# Reviewer report: Reviewer response for version 2

**DOI:** 10.21956/openreseurope.24428.r72062

**Published:** 2026-04-10

**Authors:** Virginia Howick

**Affiliations:** 1 University of Glasgow, Glasgow, Scotland, UK

The authors have addressed all my comments.

Is the rationale for developing the new method (or application) clearly explained?

Yes

Is the description of the method technically sound?

Yes

Are the conclusions about the method and its performance adequately supported by the findings presented in the article?

Partly

If any results are presented, are all the source data underlying the results available to ensure full reproducibility?

Yes

Are sufficient details provided to allow replication of the method development and its use by others?

Yes

Reviewer Expertise:

Parasitology, single-cell genomics

I confirm that I have read this submission and believe that I have an appropriate level of expertise to confirm that it is of an acceptable scientific standard.

# Reviewer report: Reviewer response for version 1

**DOI:** 10.21956/openreseurope.21399.r55289

**Published:** 2025-08-28

**Authors:** Virginia Howick

**Affiliations:** 1 University of Glasgow, Glasgow, Scotland, UK

**López-Escobar et al have provided an important study on how cell motility influences capture efficiency in T. brucei blood stages. They compared samples that were run on the 10X genomics platform at room temperature to samples from the same infection that were quenched to 0 degrees C. They found that quenching improves retention of the more motile slender form in their dataset. Generally, the paper is very clear and well presented and the method will be broadly useful to researchers using single-cell microfluidics platforms on motile and other atypical cell-types. Although the study is well designed and the results are presented clearly, I’m not fully convinced that the increased recovery of slender forms is due to reduced cell motility.**

**Major comment**

**QC thresholds impact on impact on proportion of stumpy vs slender**

**I am somewhat concerned that the loss of the slender forms in the RT condition could be due to different levels of gene expression between the two cell types and a decrease in cell quality generally, not just in slender forms. If you have lower genes per cell across samples in slender forms (as they mostly express VSG), and then you apply a high QC threshold, you will bias the sample towards removing those cells. Even if that is the case, the protocol is still useful, it just has less to do with cell motility, and more to do with overall cell quality.**

**To help clarify this, you could include the QC plots of genes and UMI per cell for each sample (like fig S1)  in order to visualise where the cut off (800 genes per cell) falls on the distribution. Does it seem like there are viable cells being removed unnecessarily? Additionally, you could visualise the genes per cell on the UMAP to see if this breaks down by cell type and by condition (RT vs quenched). Are the quenched stumpy forms also expressing fewer genes per cell in the RT condition compared to quenched? This would make me think the quenching is more a general improvement compared to motile-specific improvement. Finally, if you use a lower cut off (eg 300 genes per cell), how does that change the proportion of cell-types recovered?**

****

**Minor comments**

**DE analysis**

**A low cluster resolution was used to distinguish slender and stumpy forms; however, for the DE it might give more insight if you increased the cluster resolution to isolate the slender cluster that is majority RT cells. Within that cluster what is DE between RT and quenched? I realise the cell numbers from the quenched samples in the cluster might be too low, but worth a try.**

**Sequence saturation**

**I don’t completely follow the explanation that VSG is over saturating the sequencing in slender forms and this is a technical issue. Because 10X sequences all cells together after transcripts have their UMI and barcode, wouldn’t abundant VSG expression also decrease the number of transcripts detected in stumpy forms? Or do you think that the barcodes/UMIs within the GEM are limited? Alternatively, could it be a limitation on the cell machinery? It would be helpful to understand how deeply your samples were sequenced, i.e. did you reach sequencing saturation?**

**Page 7, first paragraph: There is no supplementary video 5 provided.**

****

Is the rationale for developing the new method (or application) clearly explained?

Yes

Is the description of the method technically sound?

Yes

Are the conclusions about the method and its performance adequately supported by the findings presented in the article?

Partly

If any results are presented, are all the source data underlying the results available to ensure full reproducibility?

Yes

Are sufficient details provided to allow replication of the method development and its use by others?

Yes

Reviewer Expertise:

Parasitology, single-cell genomics

I confirm that I have read this submission and believe that I have an appropriate level of expertise to confirm that it is of an acceptable scientific standard, however I have significant reservations, as outlined above.

# Reviewer report: Reviewer response for version 1

**DOI:** 10.21956/openreseurope.21399.r55288

**Published:** 2025-08-28

**Authors:** Igor Cestari

**Affiliations:** 1 McGill University, Montreal, Canada

This is a very well-written and nice report by López-Escobar and colleagues. The work reports single-cell experiments to improve the capture of T. brucei cells using a low-temperature fast quenching vs. room temperature and comparing results from slender vs. stumpy parasite forms. The work will be very useful in guiding experiments on T. brucei single-cell assays using microfluidics. The protocol could apply to other flagellated cells, and although differences may still exist between different cell types, the experience shared in this work will be extremely useful. 

 I noted a few points below for the authors, and I hope they take these suggestions/questions to improve the manuscript:

 1) In Table 1. Gene/cell, is this an average of all cells? Can you please indicate the variation? This will help evaluate the degree of noise and how it changes after filtering for cells with low gene counts. The same applies to the other columns.

 2) Fig 2. How does the distribution of quenched 80%:20% slender: stumpy relate to the initial distribution of slender to stumpy? How can we know that 80:20 is the expected ratio? Although the variation is lower in the quenched than in RT, it does not determine the correct distribution of slender:stumpy in the population. Having the input information would be ideal. Hence, an independent measure should be used, such as microscopic counting or flow cytometry, or perhaps another information collected by the authors, to ensure that the collected values by quenching the samples are representative of the population and if any deviation, the authors could point it out. 

 3) To my point above, the authors concluded: "quenching probably facilitated the recovery of less motile slender forms within the GEMs and thus the final sequenced population of parasites is more representative of the original population in the blood." Provide some support for this.

 4) Differences in RT1 and RT2 are very difficult to see in Fig 3A. Can you change the colours for better visualization?

 5) I wonder how many of these cells in the population represent dying cells. I am not sure if there is a specific marker that could be used, as it would be interesting to know the state of the cells. Dying cells will also have a transcriptome, but how this could affect the overall population analysis and thus add bias to experiments is unclear. Perhaps, cell cycle markers should be included in the analysis, such as those analyzed in 2C, to identify the cell cycle stages and perhaps abnormalities that could be indicative. 

 6) Does the lower right population (~4 o'clock in cluster 0) in Fig. 2A represent typical live cells in any cell cycle stage, or could those be dying cells? Why are they forming such a distinct population?

Is the rationale for developing the new method (or application) clearly explained?

Yes

Is the description of the method technically sound?

Yes

Are the conclusions about the method and its performance adequately supported by the findings presented in the article?

Yes

If any results are presented, are all the source data underlying the results available to ensure full reproducibility?

Yes

Are sufficient details provided to allow replication of the method development and its use by others?

Yes

Reviewer Expertise:

Parasitology, molecular and cellular biology, genomics, proteomics, genetics.

I confirm that I have read this submission and believe that I have an appropriate level of expertise to confirm that it is of an acceptable scientific standard, however I have significant reservations, as outlined above.

# Reviewer report: Reviewer response for version 1

**DOI:** 10.21956/openreseurope.21399.r55285

**Published:** 2025-06-27

**Authors:** Carolina Moura Costa Catta Preta

**Affiliations:** 1 University of York, York, England, UK; 2 Laboratory of Parasitic Diseases, National Institutes of Health (Ringgold ID: 106066), Bethesda, Maryland, USA

**General Comments**

 This manuscript addresses an important and often overlooked technical limitation in single-cell RNA sequencing of motile organisms. The study is well-designed and timely, particularly for researchers working with kinetoplastids and other highly motile cells. The proposed protocol modification (rapid cooling or "quenching") is clearly described and convincingly tested. I believe the paper makes a valuable contribution to the scRNA-seq methodology literature. However, several clarifications and improvements in writing and data presentation would enhance the clarity and rigor of the work.

**General Suggestions**

- Please consider depositing the processed data as a .Rds file in Zenodo as well, this will greatly improve transparency and reproducibility. Apologies if I missed it, but I couldn't find it among the other code and .Rds files.
- In the supplementary file with figure legends, include legends for the supplementary videos as well. These videos are valuable to the reader, but currently lack adequate description.

**Points for Clarification**

**1. **The authors state that earlier *T. brucei* scRNA-seq studies (e.g., references 26, 28, 32 - also 44?) achieved equal or better cell recovery rates using protocols that kept parasites on ice before 10x encapsulation. Given this, the added value of the quenched protocols described here are, of course, its ability to improve representation and reduce capture bias, rather than simply increasing overall cell recovery.

 In this regard, the authors present compelling evidence - based largely on scRNA-seq data - that quenching enhances the representation of certain subpopulations that might otherwise be underrepresented. However, the conclusions would be strengthened by including an external validation step (pre- and post- encapsulation), such as microscopy-based quantification or RT-qPCR using stage-specific markers, to confirm that the cell-type proportions observed after quenching more accurately reflect those found in vivo.

**2. **In the UMAP of merged, non-normalized RT and quenched samples, subclusters are visible within both the slender and stumpy populations. Since the authors state that no integration or batch correction method was applied to address potential technical variation, I suggest applying such methods, and maybe assess clustering stability. This could help determine whether the observed substructures reflect true biological variation or are artifacts of technical differences. Additionally, differential expression analysis between subclusters and evaluation of known marker expression could provide further insights into their biological relevance.

**3. **The methods section suggests that the authors performed unsupervised clustering, followed by annotation using marker genes from Briggs et al. (2021). Could the authors confirm whether this was a fully unsupervised approach, or if any supervised step as reclassification based on marker expression was included at any point? This clarification is especially important given the observed substructure within slender and stumpy populations in the merged UMAPs. If the clustering was truly unsupervised, additional analyses, such as testing marker gene expression across subclusters or comparing clustering outcomes with and without integration, could help evaluate whether these structures reflect biological subtypes, technical batch effects, etc.

**4. **In the section *“Quenching improves retention of slender forms”*, the final paragraph restates earlier points and could be deleted entirely without loss of clarity.

**5. **This may be a matter of writing style, but I found the directionality in the GO term discussion somewhat confusing. While the differential expression is performed as QUENCHED vs RT, the GO enrichment results are discussed as if referring to RT vs QUENCHED, without explicitly indicating the reversal. For instance, GO terms enriched in genes downregulated in QUENCHED are described as features of RT conditions. To improve clarity and help the reader follow the interpretation more easily, it might be helpful to align the direction of the discussion with the DE analysis as performed or briefly note when the direction is being inverted for narrative purposes.

**6. **While the authors report no significant enrichment of stress-related GO terms, stress-responsive changes can be subtle or missed by broad GO categories. Could they include a targeted analysis of known heat-shock or cold-shock genes to more sensitively assess whether quenching induces physiological stress?

Is the rationale for developing the new method (or application) clearly explained?

Yes

Is the description of the method technically sound?

Yes

Are the conclusions about the method and its performance adequately supported by the findings presented in the article?

Yes

If any results are presented, are all the source data underlying the results available to ensure full reproducibility?

Yes

Are sufficient details provided to allow replication of the method development and its use by others?

Yes

Reviewer Expertise:

Parasitology, Molecular biology

I confirm that I have read this submission and believe that I have an appropriate level of expertise to confirm that it is of an acceptable scientific standard, however I have significant reservations, as outlined above.
